# Assessment of the therapeutic efficacy of artemether-lumefantrine in the treatment of uncomplicated *Plasmodium falciparum* malaria in northern KwaZulu-Natal: an observational cohort study

**DOI:** 10.1186/1475-2875-11-434

**Published:** 2012-12-28

**Authors:** Charles H Vaughan-Williams, Jaishree Raman, Eric Raswiswi, Etienne Immelman, Holger Reichel, Kelly Gate, Steve Knight

**Affiliations:** 1 Umkhanyakude Health District Office, Jozini, KwaZulu-Natal, 3969, South Africa; 2 Malaria Research Unit, South African Medical Research Council, Durban, KwaZulu-Natal, 4001, South Africa; 3 Umkhanyakude Health District Office, Jozini, KwaZulu-Natal, 3969, South Africa; 4 Manguzi Hospital, Private Bag X301, KwaNgwanase, KwaZulu-Natal, 3793, South Africa; 5 Mosvold Hospital, Private Bag X2211, Ingwavuma, KwaZulu-Natal, 3968, South Africa; 6 Bethesda Hospital, Private Bag X605, Ubombo, KwaZulu-Natal, 3970, South Africa; 7 Department of Public Health Medicine, University of KwaZulu-Natal, 236 George Campbell Building, Howard College Campus, King George V Avenue, Durban, KwaZulu-Natal, 4041, South Africa

**Keywords:** *Plasmodium falciparum* malaria, Artemether, Lumefantrine, Therapeutic efficacy, Resistance markers, KwaZulu-Natal

## Abstract

**Background:**

Recent malaria epidemics in KwaZulu-Natal indicate that effective anti-malarial therapy is essential for malaria control. Although artemether-lumefantrine has been used as first-line treatment for uncomplicated *Plasmodium falciparum* malaria in northern KwaZulu-Natal since 2001, its efficacy has not been assessed since 2002. The objectives of this study were to quantify the proportion of patients treated for uncomplicated *P. falciparum* malaria with artemether-lumefantrine who failed treatment after 28 days, and to determine the prevalence of molecular markers associated with artemether-lumefantrine and chloroquine resistance.

**Methods:**

An observational cohort of 49 symptomatic patients, diagnosed with uncomplicated *P. falciparum* malaria by rapid diagnostic test, had blood taken for malaria blood films and *P. falciparum* DNA polymerase chain reaction (PCR). Following diagnosis, patients were treated with artemether-lumefantrine (Coartem®) and invited to return to the health facility after 28 days for repeat blood film and PCR. All PCR *P. falciparum* positive samples were analysed for molecular markers of lumefantrine and chloroquine resistance.

**Results:**

Of 49 patients recruited on the basis of a positive rapid diagnostic test, only 16 were confirmed to have *P. falciparum* by PCR. At follow-up, 14 were PCR-negative for malaria, one was lost to follow-up and one blood specimen had insufficient blood for a PCR analysis. All 16 with PCR-confirmed malaria carried a single copy of the multi-drug resistant (*mdr1*) gene, and the wild type asparagine allele *mdr1* codon 86 (*mdr1* 86N). Ten of the 16 samples carried the wild type haplotype (CVMNK) at codons 72-76 of the chloroquine resistance transporter gene (*pfcrt*); three samples carried the resistant CVIET allele; one carried both the resistant and wild type, and in two samples the allele could not be analysed.

**Conclusions:**

The absence of *mdr1* gene copy number variation detected in this study suggests lumefantrine resistance has yet to emerge in KwaZulu-Natal. In addition, data from this investigation implies the possible re-emergence of chloroquine-sensitive parasites. Results from this study must be viewed with caution, given the extremely small sample size. A larger study is needed to accurately determine therapeutic efficacy of artemether-lumefantrine and resistance marker prevalence. The high proportion of rapid diagnostic test false-positive results requires further investigation.

## Background

The World Health Organization (WHO) has recommended that drug efficacy be regularly assessed [1,2]. Failure to detect the emergence of anti-malarial drug resistance, could lead to a drug-resistant malaria epidemic, which would have major public health and economic consequences for an area, province and country. The most recent malaria epidemics in KwaZulu-Natal, one of three provinces in South Africa with endemic malaria, were partially attributed to unrecognized resistance to the anti-malarial therapy being used at the time [3]. Artemether-lumefantrine (AL) has been first-line treatment of uncomplicated *Plasmodium falciparum* malaria in northern KwaZulu-Natal since it was introduced in response to these drug-resistant epidemics in 2001 [4,5]. Studies should be performed to confirm the continued efficacy of AL, or provide a warning of emerging resistance, and the need to seek alternative therapy before a malaria epidemic occurs.

### Recent history of *Plasmodium falciparum* anti-malarial drug resistance in KwaZulu-Natal

Chloroquine resistance was first detected in KwaZulu-Natal in 1985 [6], and had increased by 1988 [7], leading to sulphadoxine-pyrimethamine (SP) replacing chloroquine as the first-line treatment for uncomplicated *P. falciparum* malaria in KwaZulu-Natal [4,8,9]. SP remained effective until 1996 when malaria incidence increased sharply in KwaZulu-Natal. Between 1996 and 2000 northern KwaZulu-Natal suffered increasingly severe malaria epidemics, with more than 40,000 cases reported in 2000 [4,5,10].

**Figure 1:**
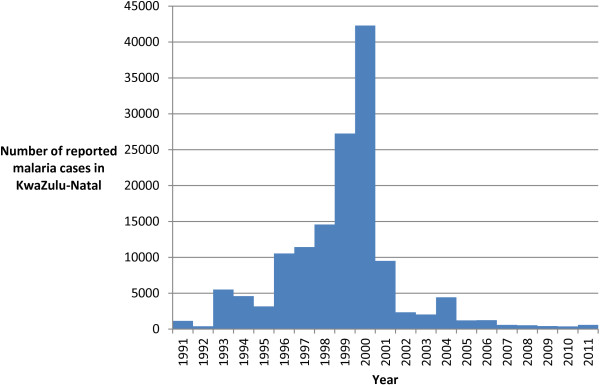
**Malaria notifications in KwaZulu-Natal from 1991 to 2001.** Source: Data 1996-2011 - KwaZulu-Natal Department of Health Malaria Control Programme; Data 1991-1995 - [3].

Only in 2000, were *P. falciparum* parasites in the region shown to have developed at least 61% (and as high as 89%, excluding those lost to follow-up) resistance to SP in a clinical efficacy study, rendering the drug ineffective in northern KwaZulu-Natal [8]. The introduction of AL as the first-line medication for uncomplicated *P. falciparum* malaria, together with the reintroduction of DDT insecticide for indoor residual house spraying in 2001, dramatically reduced malaria incidence in the area [4,5]. It has been estimated that the delay in changing first-line treatment for malaria between 1996 and 2000 was responsible for substantial morbidity and mortality, as well as contributing to the size of the epidemic [3]. Malaria notifications in KwaZulu-Natal between 1991 and 2001 are shown (Figure 1).

It has been estimated that in 2000, at the height of the epidemic, the malaria incidence amongst the exposed population in northern KwaZulu-Natal was 5,972 per 100,000 [3]. It should be noted that the malaria notification system became overloaded during these epidemics, and that the notifications were incomplete. For example during the year 2000 one clinic, Ndumo Clinic, in northern KwaZulu-Natal, saw 30,885 cases based on laboratory results, a 50-fold increase compared to 1995 [5], and equivalent to 73% of the total provincial notifications of 42,248 [10].

Artemisinin-based combination therapy (ACT) is advocated for the treatment of uncomplicated *P. falciparum* malaria because of the rapid reduction in parasite load caused by artemisisin or its derivative; the consequent reduced likelihood of resistance emerging to the partner drug; the reduction in gametocyte carriage, and rapid clinical response [11]. ACT is recommended by WHO for the treatment of *P. falciparum* malaria [12], and AL is one of the recommended combinations [2,12]. Studies of AL therapeutic efficacy in northern KwaZulu-Natal during 2001 and 2002 indicated that AL was effective for treating uncomplicated malaria in the area [4,13,14]. Since 2002, there have been no further studies of the continuing therapeutic efficacy of AL in KwaZulu-Natal, or South Africa. The WHO recommends routinely monitoring anti-malarial resistance at least every three years, and a change in anti-malarial medicine if the treatment failure proportion is equal to or greater than 10% by day 28, or the last day of follow-up, if longer than 28 days [1,12].

### Pharmacology of artemether-lumefantrine

Artemether-lumefantrine is a combination of two drugs, artemether and lumefantrine, manufactured in tablet form as Coartem® by Novartis. Each tablet contains 20 mg artemether and 120 mg lumefantrine [15]. The two drugs act in an independent but complementary manner at different stages of the parasite life cycle [16]. Artemether and its active metabolite, dihydroartemisinin, rapidly kill most circulating malaria parasites, while lumefantrine clears the remainder more slowly [16,17]. The probability of selecting parasites resistant to the partner drug, lumefantrine, is theoretically reduced due to the small parasite load remaining following activity of artemether [16,17]. Artemether is rapidly absorbed and metabolized, with a half-life of about two hours, whereas lumefantrine is absorbed more slowly and has a half-life of 3-4 days in malaria patients [16,17].

Coartem® is taken as a six-dose oral regimen over three days. The dosage depends mainly upon the weight of the patient. The dosage for persons aged 12 years or more, or younger children weighing 35 kg and above, is four tablets as a single dose at the time of initial diagnosis, four tablets after eight hours, and then four tablets twice daily on each of the following two days [15]. It is recommended that the tablets are taken with fatty food or milk to improve absorption [15,17,18].

### Choice of follow-up period for anti-malarial efficacy testing

In the 2009, WHO anti-malarial drug efficacy testing guide [1], inadequate responses to anti-malarial treatment are classified as: ‘Early Treatment Failure’ in which there are danger signs or failure to reduce parasitaemia levels by day 3; ‘Late Clinical Failure’ in which there are danger signs or parasitaemia and fever occurring between days 4 and 28, and ‘Late Parasitological Failure’ in which there is parasitaemia without fever between days 4 and 28. ‘Adequate clinical and parasitological response’ is the absence of parasitaemia on day 28 (or day 42 for longer acting drugs), irrespective of axillary temperature, in patients who did not previously meet any of the criteria of early treatment failure, late clinical failure or late parasitological failure. For drugs with a half-life of less than seven days, such as artemisinin and lumefantrine, evaluation of clinical and parasitological response up to 28 days is recommended [1]. For those with longer half-lives such as mefloquine (three weeks [19]) and piperaquine (two to three weeks [20]), a follow-up of 42 days is recommended [1,12,21].

Although there has been no anecdotal evidence of resistance to AL in KwaZulu-Natal since its implementation, artemisinin resistance, characterised by slow clearing of parasite has been confirmed in South East Asia [22], and suggested in Kenya [23]. Previous research by Roper and colleagues demonstrated that SP resistance spread to southern Africa from East Africa [24]. In neighbouring Mozambique increase in prevalence of molecular markers associated with lumefantrine resistance since initial use of AL suggest the need for continued surveillance for the emergence of resistance to the drug [25]. The primary objective of this study was to screen for late AL clinical failure, the first indication of emerging resistance to AL in South Africa.

Requiring patients to return a clinic six or seven times in one month for assessment requires considerable resources and the risk of drop-out from the study is high. A single follow-up assessment at 28 days was therefore chosen which required a patient to return only once. PCR is recommended by the WHO to distinguish between *P. falciparum* recrudescence and re-infection seven days or more after treatment in areas of both low to moderate, and high, transmission [1].

### Molecular markers of malaria resistance

According to the 2002 WHO report [26], molecular markers may assist in determining resistance and provide an early warning of developing drug resistance before it becomes clinically apparent. Markers of resistance have been validated for a number of monotherapies, including chloroquine [27] and lumefantrine [28]. As the genetic basis for artemisisin resistance is not known, efficacy of AL is assessed by analyzing molecular markers for lumefantrine resistance. Certain molecular markers have been linked with resistance to lumefantrine, the partner drug in AL, namely the *P. falciparum* multidrug resistant (*mdr)1* gene copy number [28], and the *mdr1*86N allele [29,30]. Multiple copies of the *mdr1* gene has been linked with lumefantrine resistance in Southeast Asia [28], while mutations at the codon 86 of the *mdr1* gene modulate lumefantrine efficacy [31].

Storage of blood samples on filter paper for future testing as new molecular markers become available is recommended [26].

### Ethical issues

The study was approved by the University of KwaZulu-Natal Biomedical Research Ethics Committee, and by the Health Research Committee of the KwaZulu-Natal Department of Health.

## Methods

The study population included symptomatic persons presenting to health facilities, diagnosed with uncomplicated *P. falciparum* malaria in Umkhanyakude Health District, northern KwaZulu-Natal, using the *P. falciparum* malaria rapid diagnostic test (First Response, malaria antigen *P. falciparum* (HRP2) detection rapid card test manufactured by Premier Medical Corporation Limited, Kachigam, Daman (UT) 396215, India).

### Inclusion criteria

Symptomatic patients aged from five years to 69 years, self presenting to health facilities, diagnosed with uncomplicated malaria in Umkhanyakude Health District between January and May 2012, were invited to participate in the study.

### Exclusion criteria

Patients with the following danger signs or symptoms of severe malaria: unable to drink; vomiting everything; a convulsion during previous seven days; lethargic or decreased level of consciousness; unable to stand or sit [32], were excluded. Pregnant women, patients aged less than five years and more than 69 years, and patients treated for malaria during the previous two weeks were also excluded.

### Information provided

At recruitment patients were provided with an information sheet in English and *isiZulu* detailing the purpose of the study*,* which was also explained verbally. Patient queries were answered, after which they were invited to provide written consent.

### Investigations

Finger-prick blood spots blotted on to Guthrie 903 filter paper cards (Munktell GmbH, Barenstien, Germany), and blood samples were collected from all participants for molecular analysis and malaria microscopy. The patient was then asked to return in four weeks for repeat malaria film microscopy and blood spot collection, with the offer of ZAR50 (US$5.79) in travelling expenses upon return. RDT was not performed at follow-up due to persistence of histidine-rich protein, HRP-2, in patients for as long as 28 days after parasite clearance [14,33]. Blood spots taken by nurses were sent to the investigator at the local hospital. These were then collected by the Principal Investigator, usually twice per month, and posted to the researcher performing the molecular analysis more than 400 km away.

Thick and thin blood films were prepared according to the National Health Laboratory Service standard operating procedure for processing specimens for malaria parasites [34]. Slides were stained using the Rapid modified Wright-Giemsa stain (Rapidiff); thin films being fixed with methanol before staining. Parasitaemia was calculated from the percentage of red cells containing malaria parasites observed in 10 microscope fields using the 100x lens.

Parasite DNA was extracted from all blood spots using the QIAamp DNA Mini Kit (QIAGEN, Whitehead Scientific). The extracted DNA was then subjected qPCR and nested PCR analysis to confirm the presence of *P. falciparum* parasites [35,36].

All samples confirmed as *P. falciparum* positive by PCR, were subjected to mutational analysis to detect the prevalence of molecular markers linked with resistance to lumefantrine, (*mdr1* gene copy number amplification) and chloroquine (mutations at *mdr1* codon 86, [27] and codons 72 to 76 of the chloroquine resistance transporter (*crt*) gene) [37]. At the follow-up visit, the patient was clinically assessed, and a further finger prick blood sample taken for *P. falciparum* PCR and blood film.

## Results

**Figure 2:**
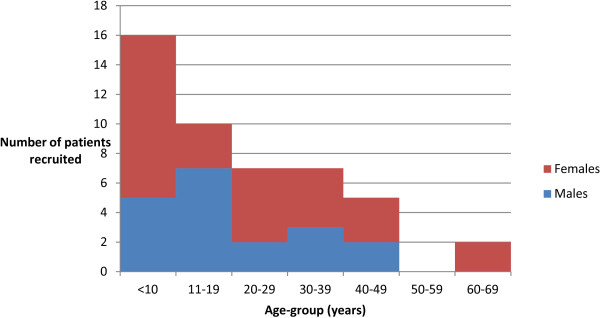
Age and gender of patients with malaria RDT-positive recruited from Umkhanyakude Health District, January to May 2012 (N=47).

A total of 49 patients with a diagnosis of malaria based on a rapid diagnostic test were enrolled in the study. Two patients did not have their age recorded. The age range of the remaining 47 patients was 2 – 69 years; median 15 years, and mean 21.1 years. The largest group comprised those less than 10 years of age (Figure 2). Four patients were less than minimum age of five years stipulated in the study protocol. Their treatment, however, was identical to that in the National Treatment Guidelines [38], and they were included in the analysis.

### Confirmation of *Plasmodium falciparum* malaria by PCR

**Figure 3:**
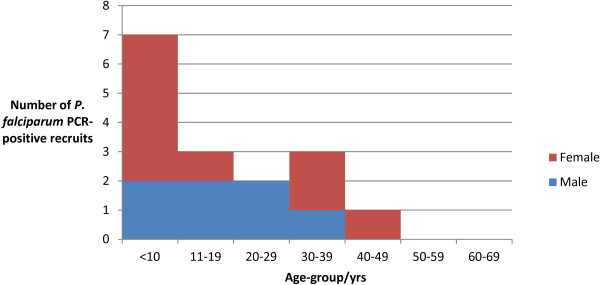
**Age and gender of patients with *P. falciparum *malaria confirmed by PCR recruited from Umkhanyakude Health District, January to May 2012 (N=16).**

Only 33% (16/49) patients were confirmed to have *P. falciparum* malaria by PCR (Figure 3). Closer inspection of the RDTs revealed that frequently too much blood had been used, rendering the test virtually impossible to interpret. The age range of those confirmed with *P. falciparum* was 2 - 40 years with median age 14.5 years and mean 16.8 years. Nearly half (7/16) of those with PCR-confirmed malaria were aged less than 10 years. Results for the *P. falciparum* PCR positive patients are summarized in Table 1.

**Table 1:** **Results for patients with PCR confirmed *Plasmodium falciparum *malaria at enrolment**

**Age**	**Gender**	**Travel 60 days prior to illness**	**Temp**	***mdr1*copy number (lumefantrine sensitivity)**	***mdr1*N86Y gene (chloroquine sensitivity)**	**Chloroquine resistance transporter gene*crt*K76T codons 72-76**	**Follow-up day**	**Follow-up*P. falciparum*malaria PCR**
40	Female	Mozambique	36.5	1	Asparagine (*mdr1* 86N)	CVMNK	28	Negative
7	Male	Not recorded	39.4	1	asparagine	CVMNK and CVIET	191	Negative
32	Female	None	36.4	1	asparagine	CVIET	168	Insufficient sample
24	Male	None	39.3	1	asparagine	CVMNK	49	Negative
9	Female	Not recorded	Not recorded	1	asparagine	CVMNK	29	Negative
31	Male	Mozambique	38.3	1	asparagine	CVMNK	32	Negative
8	Female	None	36.7	1	asparagine	CVIET	29	Negative
15	Male	Mozambique	38.0	1	asparagine	CVIET	106	Negative
20	Male	Mozambique	41.0	1	asparagine	CVMNK	127	Negative
37	Female	Not recorded	38.7	1	asparagine	CVMNK	86	Negative
6	Female	None	39.0	1	asparagine	CVMNK	28	Negative
2	Female	Mozambique	Not recorded	1	asparagine	CVMNK	NA	Lost to follow-up; returned to Mozambique
17	Male	None	38.2	1	asparagine	CVMNK	69	Negative
14	Female	None	38.0	1	asparagine	CVMNK	92	Negative
4	Female	Mozambique	38.0	1	asparagine	CVMNK	59	Negative
3	Male	Not recorded	38.0	1	asparagine	CVMNK	57	Negative

### Presence of fever

Fever (auxiliary temperature ≥37.5°C) was recorded in 64% (27/42) of all patients who were initially diagnosed with malaria and 77% (11/14) of the PCR malaria confirmed cases, while 57% (16/28) of those PCR negative had a fever. Temperature was not recorded for two malaria PCR positive patients.

### Recent travel

Six of the 16 PCR malaria confirmed cases reported having travelled to Mozambique within the previous month.

### Blood films

Due to liaison difficulties with a local laboratory, blood films were only obtained in 15/49 recruited patients. Of these, four were PCR-confirmed malaria samples, of which one was *P. falciparum* positive by microscopy with a parasitaemia of 0.25%. All other 14 blood films were microscopy negative for *P. falciparum*.

### Molecular markers

All 16 PCR *P. falciparum* positive samples collected at enrolment had a single copy of the *mdr*1 gene and carried the wild type asparagine allele at codon 86 of the *mdr1* gene (*mdr1* 86N) (Table 1). Results for 14 of the 16 samples were *crt* 72-76 genotyped. Ten samples carried the wild chloroquine sensitive haplotype (CVMNK), three the pure mutant haplotype (CVIET) associated with chloroquine treatment failure, while one carried both wild and mutant alleles. Two samples proved inadequate for *crt* genotyping.

### Follow-up

Only 14% (7/49) of the patients returned for follow-up, of which six were *P. falciparum* PCR positive at enrolment. Malaria control personnel tracked down and obtained blood specimens for follow-up PCR from a further nine non-returning PCR-confirmed patients, at varying intervals from four weeks following recruitment. Of the initial PCR positive cohort, 14 were found to be negative at follow up, one sample contained insufficient blood for testing and one patient was lost to follow-up.

## Discussion

The primary aim of this study was to assess the therapeutic efficacy of AL, the current first-line treatment of uncomplicated *P. falciparum* malaria in northern KwaZulu-Natal. The study was hampered by a scarcity of diagnosed malaria cases. Of 49 patients enrolled in the study on the basis of a positive *P. falciparum* rapid diagnostic test, only 16 were subsequently confirmed to have *P. falciparum* malaria by PCR analysis.

Amplification of *mdr1* gene copy number associated with lumefantrine resistance in South East Asia was not detected in this study [39]. This result confirms the research findings in neighbouring Mozambique [25], where no variation in *mdr1* copy number was observed following two years of AL deployment, and supports the hypothesis that *mdr1* amplification is rare in Africa [31].

The *mdr1*N86Y mutation associated with chloroquine resistance [27] was completely absent in this study. This finding together with the high prevalence of the *crt* 72-76 wild type haplotype (CVMNK) in the study area suggests AL deployment removed chloroquine drug pressure, allowing chloroquine sensitive parasites to re-emerge as seen in Malawi [40] and Mozambique [25]. This return of parasite sensitivity to chloroquine could result in the re-introduction of chloroquine in combination with a partner drug as an anti-malarial.

All follow-up samples were PCR negative for *P. falciparum*, implying sustained AL efficacy, 11 years after it was initially rolled out in KwaZulu-Natal. On a cautionary note, the high *mdr1*86N allele prevalence is a cause for some concern. It has been suggested that increases in *mdr1*86N prevalence is the first step towards lumefantrine tolerance [30]. Sustained lumefantrine drug pressure is probably driving the selection of the *mdr1*86N allele in KwaZulu-Natal. In contrast, the removal of chloroquine drug pressure probably selected for this allele in Mozambique [25]. Given the wide use of AL in southern Africa and the high prevalence of resistance markers associated with lumefantrine resistance, close monitoring of AL efficacy and lumefantrine resistance markers is recommended to ensure effective first-line treatments are available.

### Positive study outcomes

Despite the extremely small sample, some valuable data was produced by this study.

### Blood spots for molecular analysis

In a low malaria-incidence setting, blood spot samples proved to be a good a source for molecular analysis. Collection of the filter paper samples was relatively easy and inexpensive. Once collected and correctly stored the samples were resilient to delays in collection and transport. PCR is capable of detecting malaria parasites at a density of four parasites per microlitre, whereas thick film microscopy is only reliable at a density of 500 parasites per microlitre, meaning PCR is more than 100 times more sensitive than microscopy for the diagnosis of malaria parasitaemia [41,42]. PCR also has a specificity of nearly 100% [43].

Monitoring molecular markers of drug resistance, while a less rigorous method of assessing drug efficacy than *in vivo* sensitivity studies, is much less expensive and time consuming, and is a reasonable method of surveillance for emerging drug resistance [25]. The WHO recommends that in countries with very low levels of transmission, such as South Africa, studies of molecular markers of resistance should be conducted every year [1]. Molecular markers in 10 of the 14 available blood specimens indicated sensitivity to chloroquine, suggesting that chloroquine resistance may have decreased following removal of the selection pressure from using chloroquine as first line therapy. Similar findings have been demonstrated in neighbouring Mozambique and been attributed to the withdrawal of chloroquine [25].

### Study limitations

#### Enrolment procedure and administration

The incidence of malaria in the study area remained low during the study period and cases were geographically scattered, presenting to several different clinics and hospitals in Umkhanyakude District. Management and control of the patient records and specimens was difficult. The cooperation of healthcare workers from several health facilities was required and consistency of the enrolment procedure was difficult to achieve. Blood was sent by clinic nurses to the local hospital laboratory who declined to perform blood films on many patients.

Of the 49 recruited patients only 16 were PCR confirmed malaria. The high proportion of false positive RDT results was probably mainly due to incorrect use of the test, indicating a lack of familiarity, and the need for more training. It is possible that false positive RDT results have erroneously inflated the notified malaria cases in the district for some time, and is deserving of further investigation.

#### Sample size

The study aimed to obtain a sample size of 50, which is the minimum recommended by the WHO regardless of rates of failure anticipated, in order to be representative [1]. However, this study could only include the malaria cases available. In an area which suffered severe malaria epidemics within the past 12 years, partly attributable to a lack of parasite resistance data required for upgrading antimalarial treatment policy [3], it is important to undertake regular drug resistance monitoring, or risk repeating the mistakes of the past. The last published malaria resistance studies in KwaZulu-Natal took place in 2002 [4,14], and the data in this study could be used to inform a larger study.

#### Follow-up

Despite the financial incentive offered to recompense for travelling expenses, most patients had to be tracked down by malaria control personnel at varying time intervals after treatment.

#### Use of single follow-up visit

Use of a single follow-up visit on day 28, rather than follow-up visits on days 1,2,3,7,14 and 28, as recommended by the WHO [1,26,32], meant that in the event of persistence of *P. falciparum* parasitaemia by day 28, it would not be possible to distinguish between early treatment failure, late clinical failure, and late parasitological failure. The finding of persistence of parasitaemia by day 28 would provide a motivation for a further study following the WHO protocol [1] to distinguish the degree of resistance. However, as already mentioned, persuading patients to return for six follow-up visits is not easy, evidenced by the difficulty faced in this study of obtaining even a single follow-up from patients. The single 28 day follow-up should detect most clinical and treatment failures, and seems particularly suitable for screening for late clinical failure and late parasitological failure.

## Conclusions

Determining drug efficacy, particularly as malaria transmission approaches zero, is critical, as the last remaining parasites are most likely the most resistant [44]. Since therapeutic efficacy of AL in KwaZulu-Natal had not been assessed recently, this study attempted to address the issue. Unfortunately the extremely low incidence of malaria in northern KwaZulu-Natal impacted negatively on patient recruitment. As drug efficacy data is essential to inform policy, particularly as South Africa embarks on an elimination agenda [45], every attempt to obtain robust valid resistance data must be made. Future options include larger studies across multiple sites, and the follow-up of all malaria cases at 28 days with annual molecular marker studies [1].

Although 49 patients were recruited into the study based on RDT results, only 16 were confirmed *falciparum* positive by PCR. Preliminary investigations appear to indicate that incorrect use of RDT was the principal reason for the high proportion of false-positive results. Since definitive diagnosis is a fundamental tenant of the elimination agenda, further investigation into the cause of the false-positive RDT results is indicated, and corrective measures put in place to prevent misdiagnosis.

Despite the small sample size, all samples were malaria negative at Day 28, or longer, suggesting sustained AL efficacy in KwaZulu-Natal. Support for this is provided by the absence of *mdr1* copy number amplification found in this study. However rigorous regular lumefantrine resistance monitoring is recommended given the high prevalence of the *mdr1*86N allele associated with lumefantrine tolerance and widespread use of AL in southern Africa.

## Abbreviations

ACT: Artemisinin-based combination therapy; AL: Artemether-lumefantrine; *crt* gene: Chloroquine resistance transporter gene; DNA: Deoxyribonucleic acid; HRP: Histidine rich protein; *mdr* gene: Multidrug resistance gene; *pfcrt* gene: *Plasmodium falciparum* chloroquine resistance transporter gene; PCR: Polymerase chain reaction; RDT: Rapid diagnostic test; SP: Sulphadoxine-pyrimethamine; WHO: World Health Organization.

## Competing interests

The authors declare that they have no competing interests.

## Authors’ contributions

All authors have reviewed the final draft and agreed to its submission. CHVW initiated the research project, wrote the protocol, co-ordinated the research, collated the data, and composed most of the research report. JR contributed to study design, performed the PCR and molecular analysis, and assisted with editing of the manuscript. ER participated in the study design and supervised Malaria Control Personnel for the follow-up of non-returning patients. EI co-ordinated the recruitment of patients at local clinics, the collection of samples, and was responsible for overseeing clinical care of recruited patients. HR co-ordinated local recruitment of patients, collection of samples, and was responsible for overseeing clinical care of recruited patients. KG reviewed the study protocol, co-ordinated local recruitment of patients, and was responsible for overseeing clinical care of recruited patients. SK assisted with the development of the protocol and the write-up of the report of the research.
